# The Robot Selection Problem for Mini-Parallel Kinematic Machines: A Task-Driven Approach to the Selection Attributes Identification

**DOI:** 10.3390/mi11080711

**Published:** 2020-07-22

**Authors:** Cinzia Amici, Nicola Pellegrini, Monica Tiboni

**Affiliations:** Department of Mechanical and Industrial Engineering, University of Brescia, via Branze, 38, 25123 Brescia, Italy; nicola.pellegrini@unibs.it (N.P.); monica.tiboni@unibs.it (M.T.)

**Keywords:** robot selection criteria, parallel kinematic machines (PKM), task-driven design, robot kinematics and dynamics, industrial robotics, biomedical devices, mini-manipulators

## Abstract

In the last decades, the Robot Selection Problem (RSP) has been widely investigated, and the importance of properly structuring the decision problem has been stated. Crucial aspect in this process is the correct identification of the robot attributes, which should be limited in number as much as possible, but should be also able to detect at best the peculiar requirements of specific applications. Literature describes several attributes examples, but mainly dedicated to traditional industrial tasks, and applied to the selection of conventional industrial robots. After a synthetic review of the robot attributes depicted in the RSP literature, presented with a custom taxonomy, this paper proposes a set of possible requirements for the selection problem of small scale parallel kinematic machines (PKMs). The RSP is based on a task-driven approach: two mini-manipulators are compared as equivalent linear actuators to be integrated within a more complex system, for the application in both an industrial and a biomedical environment. The set of identified criteria for the two environments is proposed in the results and investigated with respect to working conditions and context in the discussion, emphasizing limits and strength points of this approach; finally, the conclusions synthesizes the main results.

## 1. Introduction

Scientific research on the Robot Selection Problem (RSP) in industrial applications has widely evolved in the last years. Since the first works in the end of the seventies, the number of robotic devices available on the market for different purposes has grown, and the number of methods to support the decision maker in the selection process among devices has increased accordingly [1,2,3,4]. Those methods basically represent objective and repeatable strategies for ranking some attributes of the different solutions, expression of robot performance characteristics or economic evaluations.

Khouja and Offodile [2] proposed an exhaustive taxonomy of the main RSP models, classifying them into five categories: (i) Multi-Criteria Decision Making models (MCDM), (ii) production system performance optimization models, (iii) computer-assisted models, (iv) statistical models and (v) other approaches. Each category offers peculiar advantages and limits. MCDM models can process large numbers of attributes, provided that data are conveniently collected, and that the relationships between robot attributes and final objectives are clearly assessed. Production system performance optimization models are particularly suitable for single product production systems, like in assembly and machine loading applications, since they can handle robot engineering attributes and product design specifications; as a drawback, these models are less flexible than those in MCDM. Computer-assisted models partially combine the advantages of the previous categories: data collected by robot application are elaborated by an expert system, which generates a list of important attributes and desired values, and a decision model allows then to select the best option within the available set, generally according to a cost minimization rationale. In this way, the contribute of the expert supports the method also in critical use cases and the automatic selection enables the analysis of many attributes. Of course the efficiency of those models is necessarily related to amount and quality of the gathered information. Statistical models are mainly focused on identifying the trade-off between attributes, therefore are particularly suitable for applications like machine loading and assembly, in which selection is often based on engineering attributes or cost parameters. Nevertheless, those data are generally collected from technical datasheets and could partially differ from the ones occurring in the actual working conditions, so that the selected robot could at worst not satisfy the expected requirements. Finally, the last category collects approaches that cannot be classified in one of the previous classes, like combinations of the presented methods one another, or with economic analyses. Within this category, the approach introduced by Nof and Lechtman in 1982 [5] compares alternative robot work strategies, evaluating the suitability of the device and of its work method with respect to the job and considering how the robot will be operated.

In 2011, Athawale and Chakraborty stated that the selection of the most appropriate method is not as important as properly structuring the decision problem, i.e., considering relevant criteria and decision alternatives [6]. Actually, further works integrate the RSP analysis investigating in detail also the attributes that different author consider within each model, listing them in itemized form [1,3,7] or defining aggregation strategies to rearrange them into clusters [4,8]. Among these taxonomies, Liang and Wang define two categories for the robot attributes, as subjective and objective ones [4,8], whereas Koulouriotis and Ketipi identify more classes, isolating for instance technical, economical and cost attributes, but also subjective, objective, qualitative and quantitative ones [4]. Since RSP originated traditionally within the industrial field, scientific literature presents several examples of methods in manufacturing environment, from design to selection of devices or components [1,9,10,11,12,13,14,15,16,17,18,19,20]. On the contrary, examples in more specific environments or applications are occasional [19,21,22], or not investigated yet, like the robot selection for tasks in biomedical environment. Nevertheless, literature states the importance of the context, since the environment introduces constraints in the device working conditions, and in the selection criteria consequently [21,23,24]. These constraints are even more critical for small scale systems, like mini- and micro-manipulators, where the reduced dimensions of the systems tend to emphasize the effect of some undesired phenomena, like potential geometric inaccuracies and their dangerous leverage effects [24,25,26,27].

Among the robotic systems, parallel architectures are of great interest, as privileged candidates for the realization of flexible and modular devices. Parallel kinematic machines (PKMs) assure higher precision in the end effector positioning and allow to achieve better dynamic performances than serial manipulators can realize, although they offer smaller workspace, bigger overall dimensions, and involve more complex kinematic equations than serial robots. For these reasons, they are adopted where high acceleration and stiffness, with reduced inertial contributions, are strictly required.

Besides, no specific investigations on the RSP for PKMs and small-scale robotics have been performed in literature yet, and the current paper starts filling this actual gap. To fulfill this aim, two quite similar parallel mini-manipulators are exploited, as significant examples of devices presenting the main challenges of both the conditions: a parallel kinematic architecture, and small-scale dimensions. According to these considerations, after a synthesis of the selection criteria currently proposed in literature, this paper compares the Spider and Tripod PKMs in a RSP rationale, investigating potential attributes for a selection process with a task-driven approach. The robots will be evaluated in a twofold illustrative task: the integration of the mini-manipulator, as equivalent linear actuator that performs a pure translation of the mobile platform, in a more complex system, in both an industrial and a biomedical environment.

Kinematic and dynamic analyses of both the devices were already described in previous literature [28,29]: in the following section, a synthesis of their significant characteristics will be briefly presented, then a set of possible selection criteria for the two environments will be depicted in the results and investigated in the discussion. Finally, the most relevant aspects will be collected in the conclusions.

## 2. Materials and Methods

### 2.1. Selection Criteria in RSP Literature

According to literature, selection factors should consider the specific requirements of manufacturing process or, in our case, the desired task in the specific environment [8]. Once defined the relevant attributes, the most suitable approach for the selection process can be detected.

In order to identify which attributes literature suggests for the selection of mini-manipulators applied to translation tasks in industrial and biomedical environment, a thematic literature analysis was performed. The attributes that emerged from the investigated papers were rearranged within three custom-defined functional categories:

- Technical attributes, including all the factors that refer to robot performance and technical characteristics;
- Economic attributes, referring to cost evaluations;
- Management attributes, collecting all the factors related to further characteristics or services.

The use of separate categories for Technical and Economic attributes is likely the most popular in literature; on the contrary, elements classified in the current work within the Management attributes category were often labeled as independent characteristics or rearranged within other classes, such as economic, objective/subjective or qualitative/quantitative attributes. Besides, the adopted classification process did not consider the attribute name only for its assignment to one category, but where needed paid attention also to the meaning of the specific attribute according to the authors aim. For this reason, we can define the suggested categories as classes of a functional taxonomy.

For the Technical attributes category, the most common requirements are repeatability and accuracy, maximum payload, velocity, number of DoFs and reach capacity, geometrical dexterity and memory capacity, but also programming flexibility and man-machine interfaces. In the Economic class, purchase and operational costs are the most used factors, although also financial indicators like the return of investment index or the net present value were stated. Finally, the most common requirements in the Management category include vendor’s service contract and quality, supporting channel partner’s performance, training delivery period, maintainability, or compliance and inconsistency with infrastructure.

**Figure 1:**
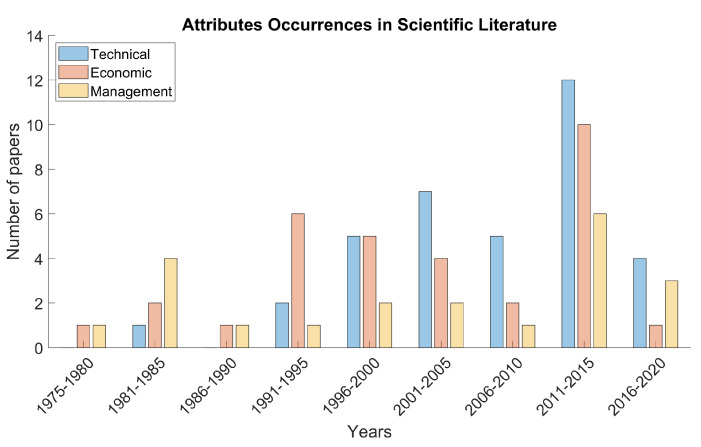
Evolution in time of the number of attributes evaluated in RSP literature, arranged by categorical areas. Data are aggregated in 5-year subsets.

A complete list of the analyzed papers is presented in Table A1, which details to which attribute classes each article contributes. Figure 1 synthesizes the same results depicting the evolution in time of the attributes occurrences in literature, by category. Data are reported in aggregated form, within five-years subsets. Even if the performed investigation does not represent a systematic literature review, the evaluated papers allow appreciating the trend of the scientific interest towards different aspects of the RSP. Analyzing the attributes as a whole, several papers propose a classification strategy devoted to distinguish between measurable and not-measurable data or quantitative and qualitative attributes; an alternative taxonomy focuses instead on the difference between objective and subjective data [8].

### 2.2. PKM Mini-Manipulators

In the following, the two compared mini-PKMs are depicted. Both the devices are no-torsion systems, and present 3 DoFs, although realized through different kinematic solutions.

#### 2.2.1. Spider Mini-Manipulator

**Figure 2:**
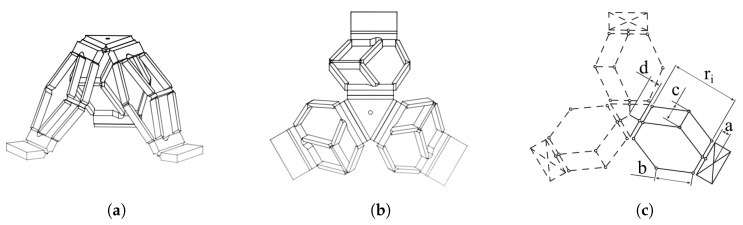
From the left, (**a**) a three-dimensional view of themini-manipulator, (**b**) the corresponding upper view, and (**c**) the basic nomenclature adopted in the kinematic analysis for the *i*th leg, with *i* = 1,…3.

**Figure 3:**
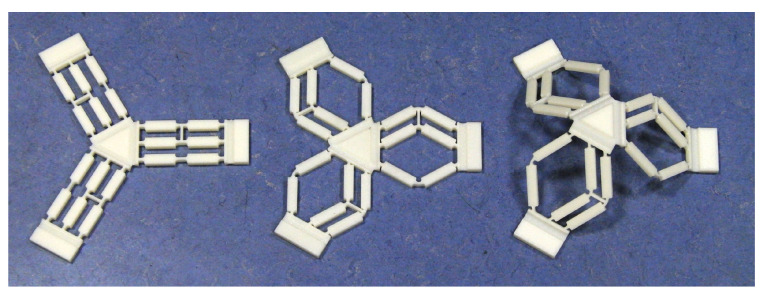
From the left, the three steps of plastic deformations at the hinges level that allows obtaining the final configuration of the mini-manipulator.

The first mini-manipulator, presented in Figure 2, is functionally equivalent to a 3-UPU system. The kinematic architecture is characterized by a strong modularity: the structure of a unit leg is repeated three times, evenly distributed around the mobile platform. The series of two four-bar mechanisms in all the legs prevents the platform from rotating around any axis, so that the platform can only perform pure translations. Besides the kinematic model, in the actual manipulator the presence of flexure hinges compensates for possible residual micro-displacements, and thanks to those hinges, the device can be also generated from a planar structure, by applying consecutive plastic deformations at the hinges level. Figure 3 depicts the deformation stages required to obtain the final configuration.

**Figure 4:**
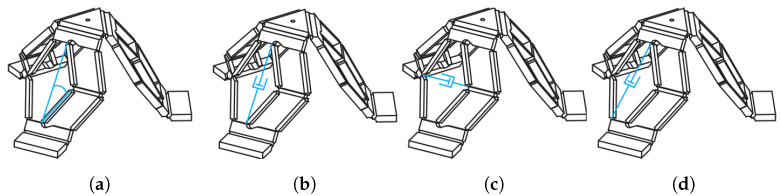
From the left, four alternative actuation strategies: (**a**) a rotative actuation, and a linear actuation between (**b**) radially aligned joints, (**c**) horizontally aligned joints, and (**d**) not aligned joints.

Given the three DoFs of the PKM, various actuation strategies can be adopted. Figure 4 presents in a synthetic way the four possible actuation solutions for each leg.

Considering the homogeneous matrix approach of Legnani et al. [30,31], the column vector S describing the pose of the mobile platform and the column vector Q of the $q_{i}$ joint parameters can be defined. Referring to the nomenclature introduced in Figure 2c, direct and inverse kinematic analyses can be easily expressed with respect to the parameter $r_{i}$. Though, different equations can describe the relation between $q_{i}$ and $r_{i}$, according to the implemented actuation strategy. For each actuators configuration, Table 1 collects the relation of the geometrical transformation between the joint parameter $q_{i}$ and $r_{i}$, and the singularity conditions of the system, in which the determinant of the Jacobian matrix vanishes.

**Figure 5:**
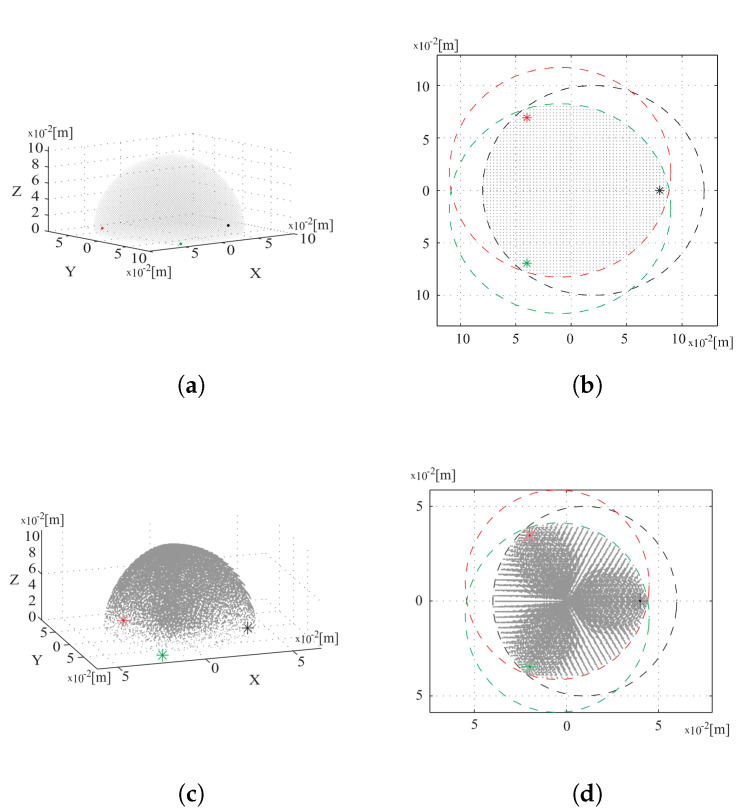
Workspace of the first mini-manipulator, with the feet of first, second and third leg in black, red and green respectively. From the left, three-dimensional and upper vision of the workspace in the ideal case for (**a**,**b**), and as evaluated according to hinges constraints for (**c**,**d**).

Independently from the actuators configuration, the system workspace can be evaluated as the intersection of the volumes that each leg, properly unburdened by constant offsets, can reach. Nevertheless, this workspace should be considered just a theoretical locus, since it grounds on an ideal model; for instance, the actual workspace would be reduced whether considering the constraints introduced by the physical limits of the flexure hinges to bending before yielding. Figure 5 presents a comparison between the theoretical workspace and the workspace evaluated under the hypotheses of hinges in homogeneous PTFE material (Young modulus equal to $500\times 10^{3}\;\left[\mathrm{MPa}\right]$, $\sigma_{Yield}$ to 50 [MPa]), and bending beam 0.2 [mm] thick and 3 [mm] wide.

#### 2.2.2. Tripod Mini-Manipulator

**Figure 6:**
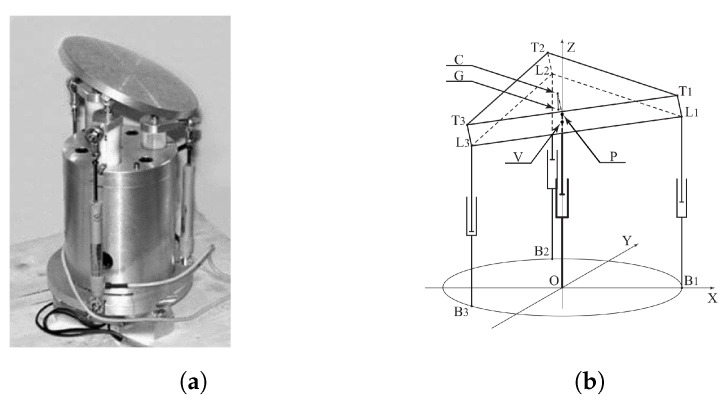
From the left, (**a**) a first prototype of the mini-manipulator in a three-dimensional view and (**b**) the functional scheme with the basic nomenclature.

The second device, depicted in Figure 6, is a 3-PSP spatial parallel mechanisms.

Actuation is provided by three piezoceramic systems, which assure by construction the functionality of an equivalent piston, since the piezoactuator within each element is protected from high pulling or shear forces by a dedicated decoupling system [32]. Although the manipulator presents three actuators for three DoFs, its kinematic model could reveal an hyperstatic condition at the connection level between each leg and mobile platform. In fact, if all the involved bodies are considered rigid and not deformable, an uneven actuation on the legs would require an increment in the distance among the mobile edges of the links (the *L* points of Figure 6b). The actual device presents flexure hinges between each piston and the mobile platform, which allow for micro-displacements; according to this, three fictitious slider constraints, coinciding with the spherical joints between each piston and the platform, were added to the model, to solve the critical conditions.

According to the Tilt and Torsion approach, the machine kinematics can be described thanks to three independent parameters, here chosen as the mean value of the pistons quotes, representative of the platform translation, and the two angles ψ and θ, defined as the corresponding Euler angles, describing the platform orientation [33,34]. Actually, the main difference between Euler and Tilt and Torsion approach lies in the definition of the third angle ϕ, which in the latter method is replaced by σ−ψ. Since the translation is an independent parameter, the device kinematics can be totally defined once the coordinates *x* and *y* of the platform center of gravity G are identified with respect to the angles ψ and θ. Referring to Figure 6b, V represents the projection of G on the platform lower surface, evaluated normally to the platform width; because of this geometrical relation, kinematics can be equivalently described with respect to V. Table 2 describes the characteristic equations of the system with respect to different assembly conditions of the device, whereas the horizontal offset allowed to the point V of the platform (see Figure 6b) with respect to the central axis in terms of ψ and θ is described in the Equation (1).

(1) $$v=\frac{1-\cos\theta }{4\cos\theta }\sqrt{\cos2\theta \cos6\phi -\cos6\phi +\cos2\theta +3},\;v\in [\frac{\cos\theta -1}{2};\frac{\cos\theta -1}{2|\cos\theta |}]$$

## 3. Results

Comparing industrial and biomedical fields, several differences arise, such as in safety- and security-related requirements for the final system and all its components. The expected characteristics of the chosen device strictly depend on the analyzed task, like the possibility to perform a pure translation in the current case. Nevertheless, this element is not sufficient to completely define the RSP; as a matter of fact, the characteristics of the context, given by a proper description of the complex mechanism and its final working condition, become fundamental as well. Indeed, a final mechanism performing robotic rehabilitation of human subjects and a robot for telesurgery would provide different constraints, although both the systems operate within a biomedical environment. Analogous situation can be detected also in industrial environment, comparing for instance a robot for food manipulation and a device for high loads handling in foundries. For this reason, a multi-purpose set of possible robot attributes was identified for the RSP. Table 3 reports the proposed attributes; in the table, requirements are arranged in the three previously introduced categories.

Among the listed attributes, besides traditional factors some dedicated items were introduced, such as the kind of actuation of the system, defined as actuation type, or the compliance to non-idealities, i.e., the ability of the robot to react to unexpected phenomena, like forces introducing loads on the robot, along not allowed movement directions. A synthetic indicator for the evaluation of the volume performance was also depicted as the ratio between the robot workspace and the overall robot dimensions, but also the maximum acceptable temperature range was included as indicative of the working conditions. Among the management attributes, the factor software and services was introduced, to collect additional elements such as the performance of the software for data acquisition, storage and analysis, or further benefit services.

## 4. Discussion

The analysis of the RSP literature suggests an interest of researchers towards different aspects of the robot involved in the process selection. Referring to Figure 1, economic attributes appear in at least one paper for all the 5-year subsets, although with an oscillatory trend. Management attributes are described in scientific works of all the subsets too, and they gained particular attention in the last decades. The data distribution for technical attributes revealed great research interest from the late nineties of the last century. Within each subset, the disparity among occurrences numbers for the different categories depicts that at least some of the analyzed papers did not include factors for all the three classes. The proposed taxonomy does not detect differences between quantitative and qualitative factors, measurable and not-measurable parameters, or objective and subjective attributes, but this classification helps structuring the attributes definition problem according to a functional rationale. This strategy is particularly suitable for the proposed illustrative task, since the need of flexibility required by different application environments represents a crucial issue; in this sense, actually, a functional approach allows overcoming the limits introduced by a pure task-oriented formulation.

According to this rationale, a wide set of attributes has been identified, and the factors have been rearranged in the three proposed categories. Nonetheless, a large number of attributes can overwhelm the decision maker, increasing the information processing burden [2]. For this reason, the decision maker is expected to select among the attributes set the most relevant requirements to include in the further analysis steps, according to personal preferences, company’s philosophy or other importance ranking strategies.

Compared to the criteria traditionally adopted in literature, the proposed technical attributes well fit micro- and mini-manipulators. As a matter of fact, the compliance of the system, as its ability to compensate for non-ideal phenomena, is a precious characteristic for small-scale devices; for instance, small geometric errors, as a not accurate location of a flexure-hinge rotation axis, could easily translate into unevenly distributed loads or unexpected and not-negligible force contributes. In the same way, dimensional variations due to temperature gradients could generate undesired effects; for this reason, temperature range and material should be among the most relevant attributes for micro-manipulators selection. The ability to decouple applied loads, maximum allowed accelerations and workspace/robot volume ratio are on the other side fundamental factors for PKM manipulators, since their kinematic architecture is particularly sensitive to this kind of criticalities.

Although all the proposed attributes can be adopted for tasks in both an industrial and a biomedical environment, the application field influences the relevance of those factors. For instance, the training delivery period stated among the management attributes hides the concept of final user and expected technical skills of the operator. Besides, if the analyzed robot is expected to be integrated into a rehabilitation device, the usability of the machine becomes particularly relevant, since the final user could be the trained clinician, but also the patient or the caregiver [35,36]. In this context, also maintenance and vendor’s service quality assume a remarkable importance. In the same way, in a biomedical environment the software performance for local data treatment, data safe storage and reporting assumes a crucial role, as well as the compliance of robot materials, actuation type or power supply with dedicated regulations [23]. On the contrary, applications in industrial environments are more likely attracted by high accelerations, operation costs or consistency with already existent infrastructures.

Considering the two proposed mini-manipulators, both the systems allow the pure translation of the mobile platform, although the Spider system prevents by construction the mobile platform from any rotation. On the other side, the Tripod robot decouples shear loads along undesired directions sheltering the piezoelectric actuators from potentially fatal damages. Both the systems present the same number of DoFs and comparable workspace/robot volume ratios, but the Spider manipulator allows higher flexibility in the actuation strategy and realization materials. Finally, since the devices are prototypes and not industrialized products, no considerations can be taken at present about costs and vendor’s policy.

The proposed approach describes a general procedure, which provides an interpretation strategy suitable for the RSP, but that could be easily applied also to the selection problem of wider systems, like a production cell, or detailed aspects and components [37], such as mini-actuators [24] or grippers [1]. In the same way, other environments and applications [38] could be analyzed according to the same functional rationale, although we expect that a focused investigation on critical issues and peculiar characteristics of different tasks and working conditions could suggest some integration to the attributes set.

## 5. Conclusions

This paper analyzed the RSP with a task-driven approach. A thematic review of the RSP literature was performed and a functional taxonomy for the classification of the robot attributes was introduced, identifying the three categories technical attributes, economic attributes and management attributes. Two mini-manipulators were then compared, within an illustrative application: the adoption as equivalent linear actuators in an industrial and a biomedical environment. A set of possible robot attributes was presented, and investigated for the manipulators, considering final task and environments. Since the considered mini-manipulators are not commercial devices, no evaluations were performed on related economic and management attributes. The proposed approach aims providing the decision maker with a wide set of attributes, defined according to the potential requirements imposed by a specific application. In this sense, the decision maker can be considered a bias for the final result of the RSP, since his/her experience, creativity and ability of interpret the application context can heavily influence the choice of the attributes subset to evaluate. In conclusion, the selection process of the most relevant robot attributes should be performed considering not only a set of factors, but also expected task, environment and working conditions, or the final context.

## Figures and Tables

**Table 1:** For each actuation strategy, geometrical relation between $q_{i}$ and $r_{i}$, and singularity conditions for $q_{i}$.

Actuation Type	Transmission Relation	Singularity Events
(a)	$r_{i}=2\cdot b\cdot \cos\left(q_{i}\right)$	$q_{i}=\{0,\frac{\pi }{2}\}$
(b)	$r_{i}=q_{i}$	$q_{i}=\{0\}$
(c)	$r_{i}=2\cdot \sqrt{b^{2}-({\frac{q_{i}-c}{2}}^{2})}$	$q_{i}=\{c\}$
(d)	$r_{i}=2\cdot \sqrt{q_{i}^{2}-c^{2}}$	$q_{i}=\{0\}$

**Table 2:** Kinematic equations of the Tripod mini-manipulator for possible working conditions; *x* and *y* refer to the coordinates of the V point.

Angle Value	Solutions
$\theta =\pm \frac{\pi }{2}$	no solutions for the characteristic equations system
θ=π	$\{\begin{array}{c} x=-\cos(4\phi -2\sigma )\\ y=-\sin(4\phi -2\sigma ) \end{array}$
$\begin{array}{c} \sigma =0\\ \sigma =\pi \end{array}$	$\{\begin{array}{c} x=\frac{\cos\theta -1}{4\cos\theta }[\cos4\phi (\cos\theta -1)+\cos2\phi (\cos\theta +1)]\\ y=\frac{\sin\theta -1}{4\cos\theta }[\cos4\phi (\cos\theta -1)-\sin2\phi (\cos\theta +1)] \end{array}$

**Table 3:** Set of possible attributes of each category for the analyzed task in both the industrial and biomedical environments.

Category	Attributes for Industrial and Biomedical Environment
Technological attributes	DoFs, actuation type, repeatability, accuracy, acceleration, load (torques, forces) capacity, compliance to non-idealities, materials, workspace/robot volume ratio, temperature range, programming flexibility, man-machine interface, power supply
Economic attributes	Purchase costs, operation costs, return of investment
Management attributes	vendor’s service contract, vendor’s service quality, supporting channel partner’s performance, training delivery period, software and services, consistency with infrastructure

## Appendix A. RSP Literature Review

Table A1 collects the evaluated RSP literature, and the classification assigned to the investigated attributes.

**Table A1:** List of the papers included in the review of the RSP literature, and classification assigned to the investigated attributes.

Authors	Year	Selection Attributes		
		Technical	Economic	Management
Graves and Whitney [39]	1979		X	X
Knott and Getto [40]	1982		X	X
Nof and Lechtman [5]	1982	X		
Huang and Ghandforoush [41]	1984		X	X
Seidmann et al. [42]	1984			X
Nnaji [43]	1986			X
Offodile et al. [44]	1987		X	
Booth et al. [45]	1993		X	
Liang and Wang [8]	1993		X	
Cook [12]	1994	X	X	
Khouja [46]	1995		X	
Khouja and Booth [47]	1995		X	
Pandey [14]	1995	X	X	X
Baker and Talluri [48]	1996		X	
Goh et al. [49]	1996		X	
Goh [50]	1997	X		X
Braglia and Petroni [51]	1999	X	X	
Parkan and Wu [52]	1999	X	X	
Khouja et al. [53]	2000	X		
Layek and Lars [54]	2000	X	X	X
Bhangale et al. [55]	2003	X		
Chu and Lin [56]	2003	X	X	X
Bhangale et al. [57]	2004	X		
Bhangale et al. [58]	2004	X		
McCrea and Navon [59]	2004	X		
Bhattacharya et al. [60]	2005	X	X	X
Kapoor and Tak [61]	2005	X	X	
Karsak [62]	2005		X	
Rao and Padmanabhan [63]	2006	X		
Yu et al. [20]	2007	X		
Almannai et al. [64]	2008		X	
Karsak [65]	2008	X		
Chatterjee et al. [66]	2010	X	X	X
Kumar and Garg [67]	2010	X		
Devi [68]	2011	X	X	X
Koulouriotis and Ketipi [69]	2011	X	X	
Rao et al. [70]	2011	X		X
Samantra et al. [16]	2011	X	X	X
Vahdani et al. [71]	2011	X	X	
Athawale et al. [72]	2012	X		
Karsak [73]	2012	X	X	
Tao et al. [74]	2012	X	X	
Bairagi et al. [10]	2014	X	X	X
Honarmande et al. [75]	2014	X	X	
Liu et al. [76]	2014	X	X	X
Vahdani et al. [77]	2014	X	X	X
Keshavarz [78]	2016	X		X
Sen et al. [18]	2016	X		
Xue et al. [79]	2016	X		X
Wang et al. [80]	2018	X		
